# Position statement of the GMA committee “teaching evaluation”

**DOI:** 10.3205/zma001674

**Published:** 2024-04-15

**Authors:** Nicolas Haverkamp, Janina Barth, Dennis Schmidt, Uta Dahmen, Oliver Keis, Tobias Raupach

**Affiliations:** 1 University of Bonn, Medical Faculty, Office of the Dean of Studies, Bonn, Germany; 2 University of Lübeck, Department of Human Medicine Studies and Teaching, Lübeck, Germany; 3 University Medical Center Göttingen, Office of the Dean of Studies, Göttingen, Germany; 4 University of Jena, Medical Faculty, Clinic for General, Visceral and Vascular Surgery, Jena, Germany; 5 University of Ulm, Medical Faculty, Office of the Dean of Studies, Ulm, Germany; 6 University of Bonn, Medical Faculty, Institute for Medical Didactics, Bonn, Germany

**Keywords:** evaluation, teaching, teaching quality, reliability, validity, medicine, health sciences

## Abstract

The evaluation of teaching can be an essential driver for curriculum development. Instruments for teaching evaluation are not only used for the purpose of quality assurance but also in the context of medical education research. Therefore, they must meet the common requirements for reliability and validity. This position paper from the GMA Teaching Evaluation Committee discusses strategic and methodological aspects of evaluation in the context of undergraduate medical education and related courses; and formulates recommendations for the further development of evaluation. First, a four-step approach to the design and implementation of evaluations is presented, then methodological and practical aspects are discussed in more detail. The focus here is on target and confounding variables, survey instruments as well as aspects of implementation and data protection. Finally, possible consequences from evaluation data for the four dimensions of teaching quality (structural and procedural aspects, teachers and outcomes) are discussed.

## Abstract (de)

Die Evaluation der Lehre kann ein wesentlicher Motor für die Curriculumsentwicklung sein. Instrumente zur Lehrevaluation werden aber nicht nur zum Zweck der Qualitätssicherung, sondern auch im Kontext der medizindidaktischen Forschung eingesetzt. Entsprechend müssen Sie den gängigen Anforderungen an Reliabilität und Validität genügen. In diesem Positionspapier des GMA-Ausschusses Lehrevaluation werden strategische und methodische Aspekte der Evaluation im Kontext des Medizinstudiums und verwandter Studiengänge erörtert und Empfehlungen für die Weiterentwicklung der Evaluation formuliert. Zunächst wird ein vierschrittiges Vorgehen für die Konzeption und Durchführung von Evaluationen vorgestellt, danach wird vertieft auf methodische und praktische Aspekte eingegangen. Schwerpunkte hierbei sind Ziel- und Störvariablen, Erhebungsinstrumente sowie Aspekte der Implementierung und des Datenschutzes. Schließlich werden mögliche Konsequenzen aus Evaluationsdaten für die vier Dimensionen der Lehrqualität (Strukturen, Prozesse, Personen und Ergebnisse) diskutiert.

## Introduction

The evaluation of teaching is the final element of the Kern cycle and should be a key driver for curriculum development [1]. Depending on the data collection instrument and the people surveyed, evaluations can collect information about structural and procedural aspects of teaching, about the behavior of individual teachers and ultimately about student learning outcomes [2]. On the one hand, teaching evaluation is suitable as an instrument for quality assurance and improvement [3], and on the other hand, evaluation instruments are also used in medical education research. In both cases, the procedures used should meet high standards of scientific work such as sufficient reliability and validity of the data collection tools, since numerous potential confounding factors must be taken into account when collecting and interpreting student evaluation data and sometimes far-reaching consequences are drawn from evaluation results.

This position paper from the GMA Teaching Evaluation Committee discusses strategic and methodological aspects of evaluation in the context of medical studies and related study programs and formulates specific recommendations for evaluation [4] in addition to general standards for evaluation that have already been formulated. The position paper is intended to serve as a starting point for medical schools to further develop their own evaluation processes; it is also intended to contribute to the creation of a more general evaluation concept for undergraduate medical education and related courses.

## Background

The discussions of the GMA Teaching Evaluation Committee crystallized a desire for developing questionnaires that would facilitate a comprehensive evaluation of teaching across courses and locations. This would allow comparisons between medical schools – also in view of the revision of the National Competence-Based Catalog of Learning Objectives for medicine/dentistry and the upcoming reform of the Medical Licensure Act. In connection with the digitisation of teaching since the summer term of 2020, various questionnaires have already been developed by members of the committee in a corresponding pilot project, which are aimed at both teachers and students and look at different levels of teaching organisation (meso and micro levels). These forms were made available to all medical schools.

A general data collection instrument will not be able to cover all dimensions of teaching quality in the same way, so prioritizing content seems sensible here. The design of an instrument that can be used universally and does not require additional hardware and software at different sites remains a challenge. There is also a need for new survey instruments that take current developments (e.g. emphasis on communication and interprofessionalism) and special teaching settings (e.g. teaching practical skills, e.g. in skills labs) into account.

## Basic considerations

The Committee recommends the following four-step approach:

1. **Defining the objective(s) of the evaluation: **Before an evaluation is carried out, the aim of the evaluation must be clearly stated and agreed upon. This also includes defining a construct to be measured as well as considerations for the exclusion of possible confounding variables. If possible, it should be determined in advance what consequences are to be drawn from the results.
2. **Selection of data collection instrument(s): **The central step in the evaluation process is the selection of one or more suitable instruments to achieve the stated goal. This requires a decision on whether to choose an existing survey instrument with known psychometric properties or whether a new instrument must be developed. For specific purposes it may be useful to use non-validated survey forms; however, in general and from a scientific perspective, it is recommended to use published and validated survey instruments.
3. **Data collection: **In principle, it must be clarified when or during which period the data should be collected and whether data collection should be paper-based or online. In the latter case, various platforms are available, some of which can automatically send notifications and reminders to the target audience.
4. **Presentation of the results: **An appropriate approach to data analysis and presentation should be taken in order to facilitate a meaningful interpretation of the data. Analyses that go beyond descriptive evaluations should only be carried out in justified cases.

Depending on the objectives, the evaluation process should have the support of the medical school committees. Responsibilities, workflows and the implementation of consequences should be communicated transparently throughout.

## Methodological and practical aspects

In the following, individual aspects of the evaluation methodology will be discussed – in some cases with reference to the four steps mentioned above.

### 1. Defining the objective(s) of the Evaluation

The first step in the evaluation process is determining the evaluation goal. Depending on this goal, suitable survey instruments will be selected. In general, the aim of evaluation in most cases is to measure the quality of teaching and to strive for improvements on this basis. For this purpose, it is necessary to define the construct “good teaching” for the context at hand. For example, in a survey of around 800 clinical lecturers at German medical schools, “good teaching” was characterized in particular by its leading to sustainable learning outcomes among students and increased interest in the content taught [5]. 

#### Target variable(s)

Two definitions of good teaching – the Stanford criteria and the four dimensions according to Gibson – will be portrayed in greater detail here because they are widely known and easy to operationalise. In addition, data collected based on these definitions can serve as a solid starting point for ongoing curriculum development. There are of course other classifications and systems for evaluating “good teaching” as well, all of which allude to similar dimensions [6], [7]. 

The Stanford criteria [8], originally developed as part of a faculty development program, are:

1. Establishing a positive learning climate,
2. Control of the teaching session,
3. Communicating goals,
4. Promoting understanding and retention,
5. Evaluation,
6. Feedback and
7. Promoting self-directed learning.

According to Gibson [2], teaching quality is a multidimensional construct. In advance of the evaluation, it must be clarified which dimension(s) of good teaching are to be assessed:

- The structural dimension includes the overall conditions under which teaching takes place, including the teaching facilities, the basic structure of a course or the existence of a catalogue of learning objectives.
- The procedural dimension refers to the teaching sessions themselves. Quality indicators commonly used here include, for example, the punctuality of teaching staff and students, the type of interaction and the use of different teaching methods [9], [10]. A term often used in this context is the “learning climate” [11].
- The personal dimension refers to the teaching staff. In most cases, teachers have little opportunity to influence structural aspects of teaching. However, they can certainly shape teaching processes. Due to the fact that teachers play an important role in most curricula, specific survey instruments have been developed to assess the performance of individual teachers [12], [13], [14], [15]. These can also be used as part of assessments relevant to career development.
- The outcomes dimension of teaching quality refers to the measurable result of teaching. This is often also referred to as the students’ learning success (although other definitions are also possible). Examinations can be used to measure learning success; however, these only reflect the level of performance at the end of a teaching intervention and do not say anything about the actual increase of knowledge, skills and professionalism during the intervention. As an alternative to determining learning success, repeated self-assessments can also be used, the reliability and validity of which have been examined in some studies [16], [17], [18].

Students are often asked – without reference to any of the aforementioned dimensions – to provide a general appraisal of teaching, for example by awarding school grades [19]. 

Such a global rating merely corresponds to satisfaction. Due to the uniformity of the school grade system, the results suggest comparability between courses. However, satisfaction ratings based solely on school grades are particularly difficult to interpret because the construct of satisfaction is very individual. In addition, not all teaching formats that students are satisfied with are effective. Conversely, not all teaching formats that have been proven to be effective lead to increased satisfaction among students.

#### Confounding variables

Any impact of construct-irrelevant factors poses a threat to the validity of target variable measurements. The risk of confounding is particularly high if the construct to be measured has not been determined precisely enough – as a consequence, it can be difficult to distinguish between valid influences and confounding influences on measurements. 

A literature review from 2015 identified numerous factors that can impact student global assessments (e.g. school grade ratings) of courses [20]. While some of these factors (e.g. course organization, communication and feedback for students) are easily compatible with the construct of good teaching, others can clearly be described as confounding variables (e.g. gender of students, interest of students in the content taught, time of data collection). Two aspects should be particularly emphasised at this point:

- Evaluation format: In one study, online data collection was associated with a lower response rate than paper-based surveys [21], [22]. At the same time, the ratings for online data collection were more positive than for paper-based data collection.
- Voluntary participation in evaluation: If participation in the evaluation is voluntary, high-performing students may be more likely to take part; this can lead to the evaluations being more positive than if all students took part. Conversely, an obligation to evaluate (if at all possible from a legal perspective) can lead to some students not taking the exercise serious enough, thus increasing construct-irrelevant variance in the data.

Although it seems hardly possible to take all confounding variables into account or adjust for them when analysing data (because due to data protection, no personal information such as the gender of participants is collected), the people who carry out evaluations and interpret their results should be aware of these effects and, if necessary, account for them when planning an evaluation.

### 2. Selection of data collection instrument(s)

The second step in the evaluation process is the selection of appropriate evaluation tool(s). A typical target audience for teaching evaluations are students. Alternatively or in addition to this, graduates or patients can also be asked to evaluate specific aspects of the teaching. Lecturers can use self-assessments (under supervision, e.g. in faculty development programs) or peer ratings to obtain feedback on the quality of their own teaching. Finally, other existing data such as progress logs or aggregated examination results can also be used to evaluate teaching. 

Depending on the data source and the evaluation goals, different data collection formats and instruments are used: 

According to a 2021 survey, the most common format used by 97% of medical schools [23] is the questionnaire, either to be completed on paper or online. A survey among German medical schools showed that online questionnaires are used at almost all locations. Half of the schools also use paper-based questionnaires [23], [24]. There are numerous general questionnaires available for evaluating academic teaching; however, aspects specific to medical education such as bedside teaching are not always adequately taken into account. For this reason, specific instruments for assessment in medical and related courses have been developed and scientifically validated.

Many of the questionnaires used by individual medical schools aim to evaluate the quality of teaching locally. Due to the specifics of some sites which are reflected in the evaluation forms, such forms normally cannot be used across medical schools. In contrast, published questionnaires with known psychometric characteristics offer the advantage of being largely location-independent; however, according to the faculties’ own information, these are currently only used at 7% of sites [23]. Combinations of validated and non-validated forms are used by 30% of medical schools [23]. While this is possible, this should be stated clearly.

Although questionnaires can contain free text fields, in most cases the questionnaires are used to collect quantitative data. In contrast, individual interviews or focus groups are more suitable for generating qualitative data. According to the medical schools, focus groups are used in 21% and interviews in 15% of medical courses in Germany [24]. There are also course debriefings, which can be more or less structured and primarily serve to discuss specific problems and solutions.

When constructing questionnaires with scaled items, research findings on questionnaire construction must, among other things, be taken into account [25], [26].

### 3. Data collection

Aspects relevant to the implementation of evaluations include the type of data collection (online, paper-based, personal or telephone interviews), curricular integration (mandatory vs. voluntary participation) and the time of data collection. Survey instruments and mandatory vs. voluntary participation have already been discussed. This section addresses the timing of evaluations. Evaluation often takes place at the end of a lecture, a course/module or a term/ study section. While teaching staff get an ideal overview of the quality of teaching in this way, this timing can have a negative impact on the motivation of students because they do not experience the consequences of the evaluation themselves. Furthermore, data collection at the end of term can have a negative impact on criterion validity due to the delay between the survey and the subject of the evaluation which is often too long. At some sites, evaluations are therefore held in the middle of a course in order to be able to make adjustments in the current term. However, too much data collection in quick succession can lead to evaluation fatigue among students, which can have a negative impact on response rates. Methods were therefore developed that make it possible to draw generalisable conclusions from feedback from a small part of the student cohort. This method was initially only examined for satisfaction ratings [27], [28]. However, a recent study shows that a reliable assessment of student learning outcome is possible even if only around 20-30% of the students in a cohort take part in the evaluation [29].

A special feature of medical education is that – especially in courses with a clinical focus – there is usually not just one single teacher instructing the entire course. Instead, a large number of teaching staff are involved in supervising smaller groups of students. This circumstance must be taken into account when evaluating individual teaching performance, e.g. by allowing students to evaluate the teachers they have been assigned to individually and shortly after the respective session.

In order to avoid confounding by dissatisfaction with individual examination performance, an evaluation of teaching should be carried out before taking the examination – although this is often difficult to implement in reality. If the examination itself is to be subjected to an evaluation by students, separate data collection must be planned for this.

The workflow for carrying out an evaluation within a medical school, as well as the consequences of its results in the broadest sense, are also part of the implementation; due to the great importance of these aspects, separate chapters are dedicated to them.

### 4. Presentation of the results

When choosing a suitable presentation format for evaluation results, various aspects should be kept in mind:

Addressees should be able to discern the sample size, the response rate, the data distribution and any floor and ceiling effects from the presentation. 

Inferential statistical analyses should only be carried out if there is a question justifying their use. In this case the procedure should be named.

Outside scientific studies, it should be clearly stated that all analyses are exploratory.

#### Workflow: Data protection and responsibilities

Ensuring teaching quality is a central task of the dean of studies. The office of the dean of studies should therefore have the personal and technical expertise to carry out evaluations professionally and communicate the results within the medical school. On the one hand, this includes technical aspects of data collection and processing but on the other hand and above all, the competence to describe evaluation objectives and to select congruent instruments based on knowledge of the current literature.

Decentralized activities can be useful (particularly for evaluating teaching innovations in defined areas). However, data protection is of particular importance in this context, for example if the performance of individual teachers is to be evaluated. If – as is very likely in this case – there is a need to refer the matter to the respective data protection commission, the relevant deadlines must be observed. Responsibility for handling evaluation data lies either with the office of the Dean (of studies) or with a person named in the respective evaluation regulations. Conversely, it must also be ensured that the people who take part in an evaluation remain anonymous. In order to collect personal data in an evaluation project, the consent of those involved may be necessary. 

## Consequences from evaluation results

An overarching goal of teaching evaluation is to identify strengths and weaknesses and – based on this analysis – to continuously improve teaching. The consequences of the evaluation results must be based on the previously defined goals. Accordingly, they can refer to teaching-related structures and processes, teaching staff and/or the teaching outcomes.

- Consequences for the structural dimension of teaching: If evaluation reveals evidence of sub-optimal teaching conditions (rooms, accessibility of digital resources, etc.), improvements must be pursued. The structural conditions of teaching also indirectly include financial resources. However, the derivation of financial consequences of evaluations is heavily debated [18] and requires that a quality-assured evaluation instrument is used and that there is comparability between different courses.
- Consequences for the procedural dimension of teaching: Depending on which optimisation needs arise from the data, measures could be taken with the aim of improving the learning climate, optimising the implementation of instructional formats or supporting learning processes in other ways (e.g. digitally).
- Consequences for the personal dimension of teaching (teaching staff): At many medical schools (82%), feedback discussions are held with teaching staff based on evaluation data [24]. In the context of a personal evaluation, the data can result in direct individual consequences such as the referral to faculty development courses. If teaching is to be given adequate weight in academic careers, promotion to a reader position can be made dependent on the results of multiple teaching evaluations by various actors (e.g. students, experts in medical education, colleagues as critical friends).
- Consequences with regard to teaching outcomes: If the evaluation shows that the desired learning objectives are not being achieved, this should have comprehensive consequences for the constructive alignment within the relevant course (i.e. are the learning objectives appropriate/are the teaching methods adequate/do the exams represent the content in an appropriate format etc.).

## Recommendations

- The assessment of teaching quality should be based on reliable data. The Committee therefore recommends that when designing and implementing teaching-related evaluations, particular emphasis should be placed on pairing the desired goals with the right data collection instruments.
- In order to enable cross-location and cross-disciplinary comparisons, validated evaluation instruments should be used whenever possible. Published evaluation instruments that take into account specific aspects of medical education and related courses (e.g. patient-centered teaching formats) are available and should be used accordingly [13], [15].

## Note

The position paper was acepted by the GMA executive board at 24-01-2024.

## Authors’ ORCIDS

- Nicolas Haverkamp: [0000-0001-7814-8425]
- Janina Barth: [0000-0003-1905-1257]
- Uta Dahmen: [0000-0003-3483-3388]
- Tobias Raupach: [0000-0003-2555-8097]

## Competing interests

The authors declare that they have no competing interests. 
